# Binocular function in the aging visual system: fusion, suppression, and stereoacuity

**DOI:** 10.3389/fnins.2024.1360619

**Published:** 2024-02-28

**Authors:** Yutong Song, Xi Wang, Meng Liao, Alex S. Baldwin, Longqian Liu

**Affiliations:** ^1^Department of Ophthalmology and Laboratory of Optometry and Vision Sciences, West China Hospital, Sichuan University, Chengdu, Sichuan, China; ^2^McGill Vision Research Unit, Department of Ophthalmology and Visual Sciences, McGill University, Montreal, QC, Canada

**Keywords:** aging, contrast threshold, interocular suppression, binocular combination, stereopsis

## Abstract

**Introduction:**

Changes in vision that occur in normal healthy aging can be seen in fundamental measures of monocular vision. However, the nature of the changes in binocular vision with age remain unclear.

**Methods:**

A total of 28 older (53–66 years) and 28 younger adults (20–31 years) were enrolled in this study. We performed a battery of tests to assess differences in monocular contrast thresholds and various binocular visual functions including dichoptic masking weight and strength, the binocular balance point for fused stimuli, and stereoacuity in the aging and control groups.

**Results:**

Aging significantly increased monocular contrast thresholds (*p* < 0.001). Although this suggests that aging reduces the effective “input gain” to vision, we also found a significantly elevated contribution of those weaker signals to interocular suppression (*p* < 0.001). Consequently, there was no significant net difference in the strength of interocular suppression (*p* = 0.065). We did not find a significant difference of absolute balance point between the two groups (*p* = 0.090). Lastly, the mean stereoacuity was worse in the older group compared to the younger group (*p* = 0.002).

**Discussion:**

Our findings confirm previous results showing differences in contrast sensitivity and stereoacuity with aging. Furthermore, we find a change in interocular suppression that is a possible consequence of the change in contrast sensitivity. It is suggestive of a cortical system that maintains a homeostatic balance in interocular suppression across the lifespan.

## 1 Introduction

In the coming decades, the proportion of older individuals in the global population is set to increase significantly (Lutz et al., 2008; United Nations, Department of Economic and Social Affairs, Population Division, 2010). In 2020, there were estimated to be 727 million people aged over 65 years old. This population is projected to increase more than double by 2050 (Collaborators GBDDF, 2022). Aging has profound effects on the different systems in the body (Ho and Dreesen, 2021; Fulop et al., 2023). Changes in vision with aging have a significant impact on quality of life (Carabellese et al., 1993). These visual deficits are associated with both structural and functional changes of visual system (Bergholz et al., 2019). The most familiar of these is presbyopia. The crystalline lens gradually stiffens as we age. This results in a decreased accommodative function and reduced near visual acuity (Papadopoulos and Papadopoulos, 2014). Another ocular change would be the development of cataracts, that cloud the lens and so degrade the retinal image. Previous studies have distinguished such “optical” changes that occur before the transduction at the retina from “neural” changes that affect the transduction process and the processing of the subsequent signals (Green, 1970; Elliott, 1987; Kersten et al., 1988).

When considering neural changes, several monocular visual functions have been found to be affected by aging (Erdinest et al., 2021), such as contrast sensitivity (Elliott, 1987), dark adaptation (Jackson et al., 1999), and color vision (Ichikawa et al., 2021). Contrast sensitivity is associated with various everyday visual functions, for example accounting for face recognition and stair identification (West et al., 2002). Previous studies have demonstrated that the deterioration of monocular contrast sensitivity usually begins in higher spatial frequencies (where refractive changes may play a larger role) around the age of 40–50. The deficit then extends down to a wider range of spatial frequency in later life (Derefeldt et al., 1979; Owsley et al., 1983; Liutkeviciene et al., 2013).

In this study, we are interested in changes in measures of binocular visual function in the aging population. In normal binocular vision, the inputs from each eye are combined in the early stage of cortical processing (Meese et al., 2006). Having different viewpoints, the images received from the two eyes are not the same (Zhang et al., 2011; Wang et al., 2019). The similar features received from the two eyes fuse together, whereas dissimilar features are suppressed to form a single binocular percept (Blake and Wilson, 2011; Spiegel et al., 2016). The inputs from the two eyes may not be equally balanced, leading to preference for one eye over the other both in the weight of the combination of fusible features and in the decision as to which eye’s dissimilar input will be suppressed (Georgeson and Wallis, 2014; Spiegel et al., 2016). The binocular interactions between the inputs from the two eyes unlock one ability that cannot be performed monocularly: stereopsis, the perception of depth from binocular disparity. Different aspects of these processes of binocular combination and interocular suppression can be investigated through a variety of psychophysical tasks (Blake and Wilson, 2011; Baldwin and Hess, 2018). These binocular functions have been found to be impaired in patients with glaucoma (Maiello and Kwon, 2023), anisometropia (Levi et al., 2011), and amblyopia (Spiegel et al., 2016; Zhao et al., 2017; Mao et al., 2020). Although the effects of aging on binocular visual processing have also been investigated (Spear, 1993; Speranza et al., 1995), the changes that occur with age are still a matter of debate.

Conflicting results have been reported from studies looking at the effect of aging on interocular suppression. Ukai et al. (2003) and Norman et al. (2007) demonstrated that the magnitude of suppression was larger in the aging population, which may be due to the increased concentration of the inhibitory neurotransmitter gamma aminobutyric acid (GABA) within the aging human visual cortex (Pitchaimuthu et al., 2017; Abuleil et al., 2019). However, Karas and McKendrick (2015) reported a comparable perceptual surround suppression of high central contrast between the older and younger observers. In animal models, the strength of surround suppression was reduced in receptive field of V1 in older primates (Fu et al., 2010). In terms of binocular combination, few studies have explored how aging affects the contributions of each eye to fusion (Yan et al., 2021).

Several investigators have investigated changes in stereopsis with aging. Some of those studies have found poorer stereopsis in the older population (Brown et al., 1993; Lee and Koo, 2005). Garnham and Sloper (2006) measured the stereoacuity (minimum disparity required for depth judgments to be made) of aging individuals by using the TNO, Titmus, Frisby near and Frisby-Davis distance tests. They found that all of these tests showed a higher stereoacuity threshold in the older group compared to the controls, and the TNO test exhibited an even larger value. However, in a study using a line element stereogram, Norman et al. (2008) assessed the stereopsis in observers with age ranging from 18 to 83 years old. They found that stereopsis was similar between the older and younger groups.

Here, we have conducted a broad investigation of changes in binocular visual function in aging individuals. To eliminate possible confounding effects arising from using tasks with different stimulus parameters, we have assessed binocular combination, interocular suppression and stereopsis at a fixed moderate spatial frequency (2.5 c/deg), at which the optical changes are unlikely the main factors affecting these visual functions (Hess and Woo, 1978; Elliott, 1987), in aging and control (younger adults) groups. Furthermore, we have investigated sensory eye dominance, which reflects the imbalance in strength between the two eyes. This can be assessed for different tasks by calculating the difference between each eye’s threshold in monocular contrast detection, the difference between the dichoptic masking weight in a dichoptic masking experiment, and the difference of the contribution of each eye to the fused percept in a combination task. Previous studies in young adults have suggested that some of these sensory eye imbalances are correlated with each other (Wang et al., 2021), and that stereoacuity is significantly correlated with some aspects of sensory eye imbalance (Xu et al., 2011; Han et al., 2018; Wang et al., 2021). In this study, we further explored the correlations among sensory eye imbalance and stereopsis in the aging populations.

## 2 Materials and methods

### 2.1 Participants

A total of 56 participants were enrolled in this study. There were two cohorts: a group of 28 older adults (mean age: 58 years old, range 53–66; 18 females), and a group of 28 younger adults (mean age: 23 years old, range 20–31; 18 females). The refractive error of each participant was evaluated by the same optometrist. The subjects with an astigmatism or interocular spherical difference larger than 1.50D was excluded. The mean spherical equivalent power was −2.78 ± 1.7 D in the control group and − 0.31 ± 1.1 D in the aging group. The monocular best visual acuity or best corrected visual acuity of all participants were found to be better than 0.1 LogMAR at either near (with logarithmic near visual acuity chart) or distance (with ETDRS visual chart) viewing. Participants received appropriate spectacle correction during the study if needed. The crystalline lens and vitreous were assessed in all participants to ensure that the optic media within the pupillary zone was clear. Participants were excluded if they had any ocular diseases, such as strabismus, amblyopia, or retinal disease. This study followed the principles of the Declaration of Helsinki. Ethics approval was obtained from the Ethics Committee on Biomedical Research, West China Hospital of Sichuan University. All participants gave written informed consent before data collection.

### 2.2 Apparatus

The experiments were programmed and controlled using MATLAB R2018b (Mathworks, Natick, MA) with Psychtoolbox (v3.0.9; Kleiner et al., 2007), running on an Apple MacBook Pro. The stimuli were presented through gamma-corrected head-mounted 3D goggles (GOOVIS pro, NED Optics, Shenzhen, China). The resolution of the goggles was set at 1920 by 1,080 pixels. The displays occupied 46 by 26 degrees of visual angle. This gave a resolution of 42 pixels per degree of visual angle. The refresh rate was 60 Hz, and the maximum luminance was 150 cd/m^2^. All participants were instructed to lean on a forehead and chin rest to maintain fixation stability.

### 2.3 Stimuli and procedure

Before the tests began, participants performed an alignment task to avoid image misalignment. During this task, one vertical line segment was presented to each eye, green for the left eye and red for the right eye. The participants adjusted these two segments by using a keyboard (up, down, left, and right), until the vertex of two segments intersected. The corresponding coordinates of the two adjusted segments were then used to display the stimuli in the two eyes in the subsequent tests. Then, the participants completed a battery of tasks, the experiment design of which was similar to our previous study (Wang et al., 2021). Participants were allowed to take a break after every block and started the next one when they were ready to proceed.

### 2.4 Contrast thresholds

**Figure 1:**
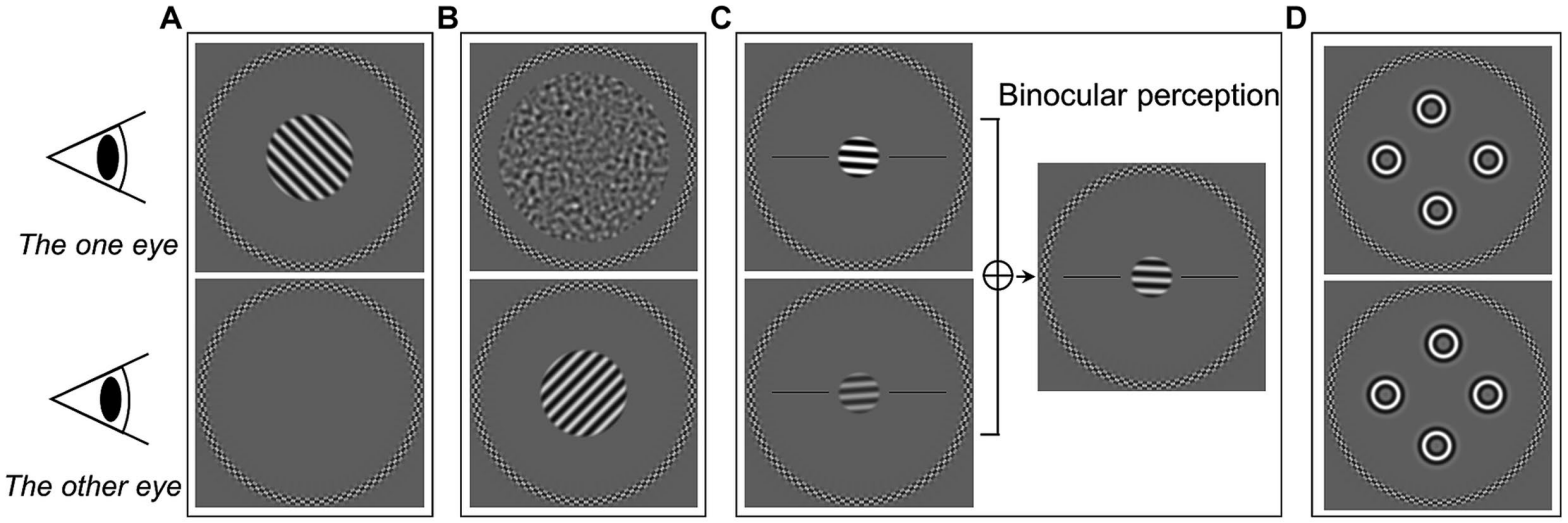
Illustrations of experimental stimuli of the five tests in the current study. **(A)** Monocular contrast threshold test. **(B)** Dichoptic masking test. **(C)** Binocular orientation combination test. **(D)** Four circles stereogram (4-C) test.

Sinusoidal gratings with a spatial frequency of 2.5 c/deg. were used in the monocular contrast thresholds tests (Figure 1A). The circular grating patches had a diameter of 3.2° of visual angle. The gratings were oriented at either −45°(left oblique) or + 45°(right oblique). The duration for displaying the grating was 480 ms in each trial. A circular frame was presented surrounding the grating location in each eye to help participants maintain a stable fusion throughout the experiments. Before adding the spatial envelope, the grating contrast was computed as root mean square (RMS) contrast. This was for consistency with the noise mask stimuli used in the dichoptic masking test. The stimulus contrast was presented in decibel logarithmic units


(1) $$\mathrm{dB}\;\mathrm{Contrast}=20\times \log_{10}\left(RMS\;\mathrm{Contrast}\right)$$

In the monocular contrast thresholds test, the target grating was presented to one eye randomly, while the other eye saw only the gray background with the circular frame (Figure 1A). A single-interval orientation identification task was used. Participants identified the orientation of the grating by pressing one of two buttons on a keyboard. The participants were given audio feedback based on whether the response was correct. The initial contrast of grating was set at −12 dB. The target contrast on each trial was controlled by a staircase algorithm. A pair of staircases (one for each eye) (As, 2019) were randomly interleaved, and followed a three-down-one-up rule with a step size of 3 dB. Therefore, they converged at the 79% correct point of psychometric function (Garcia-Perez, 1998). The test ended when each staircase reached 9 reversals or 120 trials (whichever was reached first). The test was repeated twice, and the two repetitions were combined to obtain the thresholds through psychometric function fitting (see section on *Data analysis* below).

### 2.5 Dichoptic masking thresholds

To measure thresholds under the dichoptic masking condition, target gratings (identical to those in the contrast threshold condition) were presented to one eye and masking noise to the other eye (Figure 1B). The eye which received the target was selected randomly on each presentation. The noise masks were created by bandpass-filtering white noise. The peak spatial frequency of the noise mask matched the target (2.5 c/deg). The mask had a larger diameter of 6.2°. The target grating and noise mask were presented simultaneously for 480 ms. The task was to identify the orientation of the target gratings, in the same single-interval orientation identification task as the contrast task (with the same feedback).

We used the “sideways” measurements of masking (fixing the target contrast and varying the mask) to evaluate dichoptic masking threshold (Tibber et al., 2014; Wang et al., 2021). The contrast of the target grating was fixed at a constant level for each eye throughout the test. This was 9 dB above the monocular contrast thresholds for that eye, which were obtained from the previous measurement described above. We used two staircases (one for each eye) to control the contrast of noise. The starting contrast was −36 dB. Each of the staircases was three-up-one-down, and set so that correct responses would *increase* the contrast of the masking noise. We again used a step size of 3 dB. Staircases terminated after 9 reversals or a total of 120 trials. The measurement was repeated twice. The data were combined and further analyzed to obtain the masking weight parameter and masking strength (see the *Data analysis* section below).

### 2.6 Binocular eye balance

Binocular balance was measured using a binocular orientation combination task (Wang et al., 2019, 2021). The stimuli presented to the two eyes were a pair of opposite tilted (−4° and + 4°) 2.5 c/deg. sinusoidal gratings with a diameter of 1.6° (Figure 1C). The base contrast of the grating was 45%. Seven interocular contrast ratios (1,4, 1:2, 1:√2, 1:1, √2:1, 2:1, and 4:1) were randomly selected in different trials. The contrasts shown to *both* eyes were scaled to achieve these ratios. For example: a 4:1 ratio showed a 90% contrast grating to one eye and 22.5% contrast grating to the other. Participants perceived a fused grating from the binocular combination of the gratings of opposite tilt which were presented to each eye. The participant’s task was to indicate in which direction the perceived grating was tilted. There were two black reference horizontal lines to help participants determine which side the grating was tilted toward. Each interocular contrast ratio was tested 20 times. Participant completed two repetitions of the measurements. We then combined data for further analysis to obtain the binocular balance point.

### 2.7 Stereo thresholds

We used the four circles stereogram (4-C) test (Wang et al., 2021; Atchison et al., 2022) to obtain stereoacuity thresholds. The stimuli consisted of four identical spatially-bandpass circles (Figure 1D), which had a spatial frequency of 2.5 c/deg. and a diameter of 1°. The circles were located at the top, bottom, left, and right, roughly with an eccentricity of 2° from the center. A disparity was employed to one of the circles by horizontally shifting its position in each eye in opposite directions with an equal amount of shift applied to each eye (Figure 1D). In that way, the crossed or uncrossed disparity of the circle was created. Thus, in each trial, one of the four circles would randomly appear in front of (crossed disparity) or behind (uncrossed disparity) the other three circles. The four-alternative-force choice (4AFC) method was used, where participants identified which circle had depth by pressing one of the four buttons. Audio feedback was given to indicate the correctness of the response. The initial disparity was set at 512 arc sec. We used a pair of staircases (one for each disparity direction) to control the disparity in each trial. The disparity was adjusted by following a two-down-one-up rule (converging at approximately 70% correct) with a step size of √2. Each staircase ended after 9 reversals or 30 trials, whichever was reached first. Participants completed two repetitions of this procedure. Data were combined together and the psychometric functions were used to fit to find the stereo thresholds. In addition, we measured the stereoacuity by Titmus and TNO tests with appropriate spectacles correction in both groups if necessary. During the tests, the participants were asked to order objects in depth (tell one object is in front or behind of another) to prevent the use of binocular non-stereoscopic cues (Chopin et al., 2019). To reduce the possibility that participants use monocular cues with the test, we checked their response by inverting the stereo targets and asking the participant if the target appeared in front of or behind the page.

### 2.8 Data analysis

We used Matlab R2018b (Mathworks, Natick, MA) for our analysis. The monocular contrast thresholds, dichoptic masking thresholds, and stereo thresholds were estimated through logistic psychometric function fitting with Palamedes (Prins, 2009). Thresholds were calculated at the 75% correct point for single-interval orientation identification and 62.5% for 4AFC tasks.

To fit our masking data, we used a modified two-stage model of Meese et al. (2006). Briefly, this model compromises two distinct stages of contrast gain control, one before and one after binocular combination. The response of the model to stimuli in the tested eye (right eye) at the first stage was given by


(2) $$resp_{R}=\frac{{\left(g_{R}\times C:T_{R}\right)}^{m}}{S+g_{R}\times C:T_{R}+w_{L}\times g_{L}\times C:M_{L}}$$

and the response at second stage was given by:


(3) $$\mathrm{resp}=\frac{resp_{R}^{p}}{Z+resp_{R}^{q}}$$

where $C:T_{R}$ and $C:M_{L}$ refer to the contrast of target grating (right eye) and masking (left eye) respectively. The m, p, and q are fixed model parameters set to 1.3, 8, and 6.6 respectively, which have derived from the previous works (Baker et al., 2008; Wang et al., 2021). The S and Z are fixed to 1 and 1 as descripted by the study from Wang et al. (2021). The response is calculated separately for right and left eye target conditions. The four fitted parameters are the gain in the right and left eye ($g_{R}$ and $g_{L}$), and the interocular suppression weight to right and left eye ($w_{R}$ and $w_{L}$). The $w_{R}$ represents the incoming suppression weight from mask (left eye) to right eye target condition, and $w_{L}$ is the opposite way around. The binocular response *resp* represents the expected response to the target/mask stimulus arrangement (C:T and C:M) given the fixed (m, p, q, S, Z) and fitted ($g_{R}$, $g_{L}$, $w_{R}$, $w_{L}$) model parameters. To obtain thresholds, we assume an internal response variability of unit standard deviation (allowing this to vary would simply trade off against our gain parameter) and solve for the contrast threshold that results in a *resp* of 1. We fit the model in MATLAB using the fminsearch function to minimize the root mean square error between the thresholds predicted by the model and the empirical data. These four fitted parameters are converted into logarithm value (dB), as calculated (an example of $g_{R}$):


(4) $$G_{R}=20\times \log_{10}\left(g_{R}\right)$$

We then recorded the four parameters: $G_{R}$, $G_{L}$, $W_{R}$, and $W_{L}$. The $W_{R}$ represents the incoming suppression weight from mask (left eye) to right eye target condition, and $W_{L}$ is the opposite way around. These two parameters were recorded as the “masking weight” of the two eyes for further statistic analysis.

Next, we calculated the net suppression which is the overall interocular suppression strength. This results from combining the input gain of the masked eye with the suppression weight that the target eye receives as an input from the masked eye. For example, the right eye receives a suppression from the left eye which is determined by both the left eye’s gain ($G_{L}$) and the weight of suppression that the right eye receives ($W_{R}$). The interocular suppression strength (supstr) was given by:


(5) $$\mathrm{supst}r_{R}=G_{L}+W_{R}$$

and


(6) $$\mathrm{supst}r_{L}=G_{R}+W_{L}$$

We then recorded the $\mathrm{supst}r_{R}$ and $\mathrm{supst}r_{L}$ as “masking strength” of the two eyes, and were then used in the further statistic analysis (see below).

For the binocular orientation combination task, we fit the data by using a logistic function. The psychometric function describes the proportion of left eye dominant responses at each interocular contrast ratio. The estimated midpoint of the function is defined as the point of subjective equality, at which the two eyes contribute equally to binocular combination. We regarded this interocular contrast ratio as the Balance Point (BP). Then, this BP was transformed into logarithm value (log_10_). A value of 0 indicates equal contribution of the two eyes to the binocular percept.

### 2.9 Statistical analysis

Data were analyzed in SPSS, version 27.0. The two-sided Mann–Whitney *U* tests were used to compare the difference of monocular contrast thresholds, masking weight parameter, masking strength, BP, and stereoacuity between the aging and control groups. The stereoacuities tested by Titmus and TNO were converted to logarithm values (log_2_) before analysis. Then, the log_2_ values of the 4-C test, Titmus and TNO were compared by analysis of variance (ANOVA). Spearman tests were used to determine the correlations between the binocular visual functions. The differences among the correlation ρ values were calculated using the Fisher r-to-z transformation. An alpha value of 0.05 was used to determine statistical significance. The power (1-β) of this study was 99.9, 99.6, and 91.4% respectively, based on the sample size and the results of monocular contrast threshold, masking weight parameters, and stereoacuity tested by 4-C test (calculated by G Power, version 3.1.9.4).

## 3 Results

**Figure 2:**
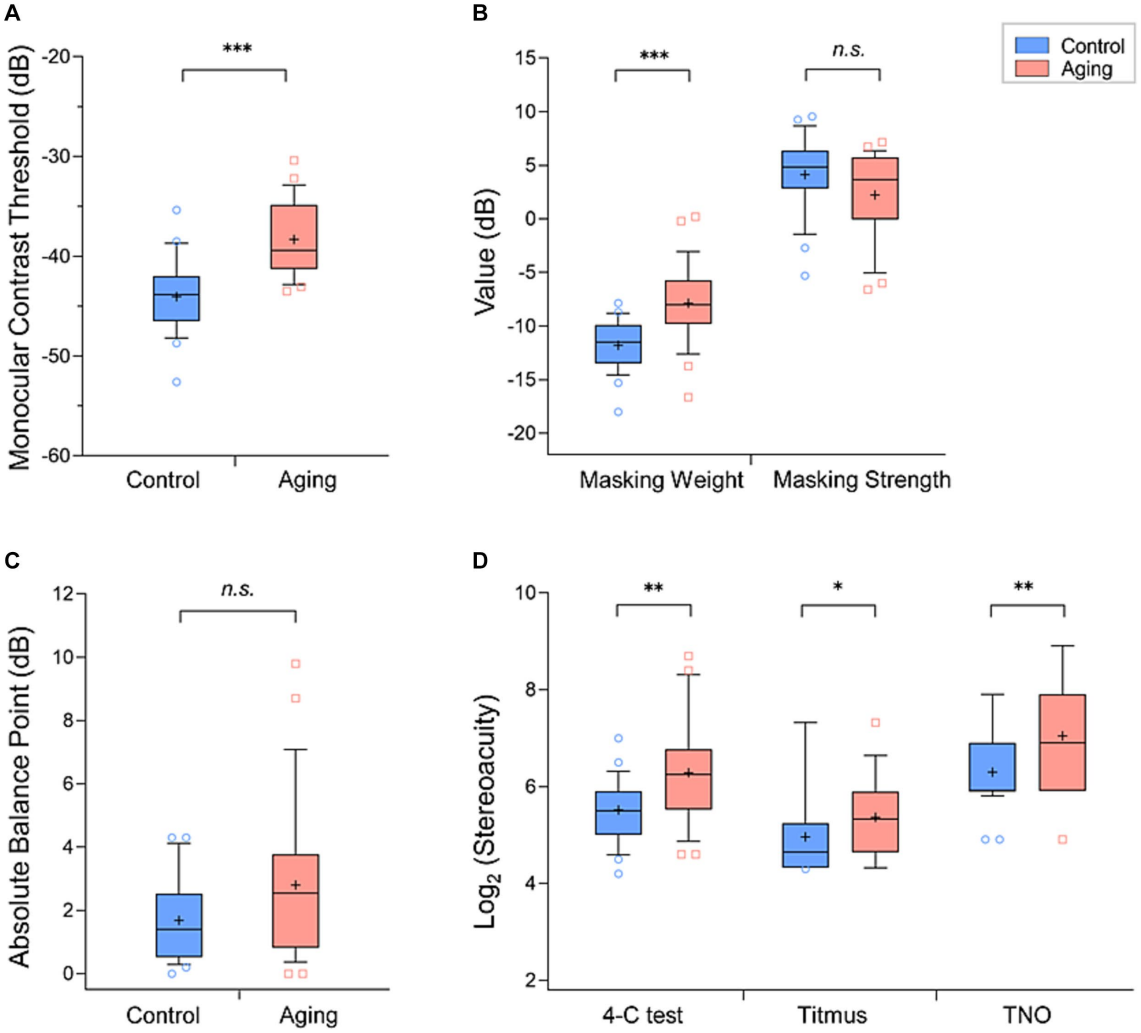
Comparison of the mean values (+) of each parameter in the aging group (*red*) and control group (*blue*): **(A)** monocular contrast thresholds, **(B)** masking weight parameters and strengths, **(C)** absolute balance point (BP), and **(D)** Log_2_ (stereoacuity). The box and whisker plots show the 10–90 percentile number in each group. The *blue* circles (control individuals) and *red* squares (aging individuals) represented the values beyond the range of the 10–90 percentile. *p* < 0.001 was marked with ***, 0.001 ≤ *p* < 0.01 was marked with **, 0.01 ≤ *p* < 0.05 was marked with *. n.s., non-significant.

We found that the mean monocular threshold of the aging group (−38.3 ± 3.6 dB, mean ± standard deviation) was significantly higher than that of the control group (−44.0 ± 3.6 dB) (*Z*_[54]_ = −5.00, *p* < 0.001, Figure 2A), meaning that the aging group is less sensitive.

In our measurements of interocular suppression, the aging group had a significantly higher masking weight parameter value (aging group: −7.9 ± 3.6 dB, control group: −11.8 ± 2.3 dB; *Z*_[54]_ = −4.52, *p* < 0.001, Figure 2B). We further calculated the interocular suppression strength by summing the input gain and the suppression weight parameters. In this comparison we did not find a significant difference between the control (4.1 ± 3.6 dB) and aging (2.2 ± 3.9 dB) groups (*Z*_[54]_ = −1.84, *p* = 0.065, Figure 2B). In binocular combination, although it appears that the balance points of the aging group scattered more widely than the control group, the difference of mean absolute balance point was not significant between the groups (control group: 1.7 ± 1.3 dB, aging group: 2.8 ± 2.4 dB; *Z*_[54]_ = −1.70, *p* = 0.090, Figure 2C).

The mean stereoacuity of the two groups obtained with the three tests are provided in Table 1 and in Figure 2D. To compare the stereoacuity measurements obtained under the various testing methods between the control and aging groups, we performed the two-way ANOVA tests. We found a main effect of group (*F*_[1,162]_ = 21.41, *p* < 0.001), suggesting that the aging group had a poorer stereo compared to the control group. In addition, the effect of testing methods was significant (*F*_[2,162]_ = 38.80, *p* < 0.001). Meanwhile, the groups × tests interaction was non-significant (*F*_[2,162]_ = 0.73, *p* = 0.486). The results of post-hoc analysis with Bonferroni corrections test showed significant differences between either of two stereo tests (*p* < 0.001, for all). The Titmus test demonstrated the smallest stereoscopic thresholds compared to 4-C and TNO tests (Figure 2D) in both groups.

**Table 1:** Mean values of stereoacuity measurement parameters obtained with four-circles (4-C), Titmus, and TNO tests.

	Four-circles (4-C)		Titmus		TNO	
	log_2_ α	linear α	log_2_ α	linear α	log_2_ α	linear α
Aging	6.3 ± 1.0	79 arc sec	5.4 ± 0.8	42 arc sec	7.1 ± 1.1	137 arc sec
Control	5.5 ± 0.7	45 arc sec	5.0 ± 1.0	32 arc sec	6.3 ± 0.8	79 arc sec

Each parameter returns a stereoacuity (indicated by α) in arc seconds, which was log_2_ converted for our analysis and reported here as the mean with standard deviation (mean ± SD). We also report the values converted back to linear stereoacuity in arc seconds.

**Figure 3:**
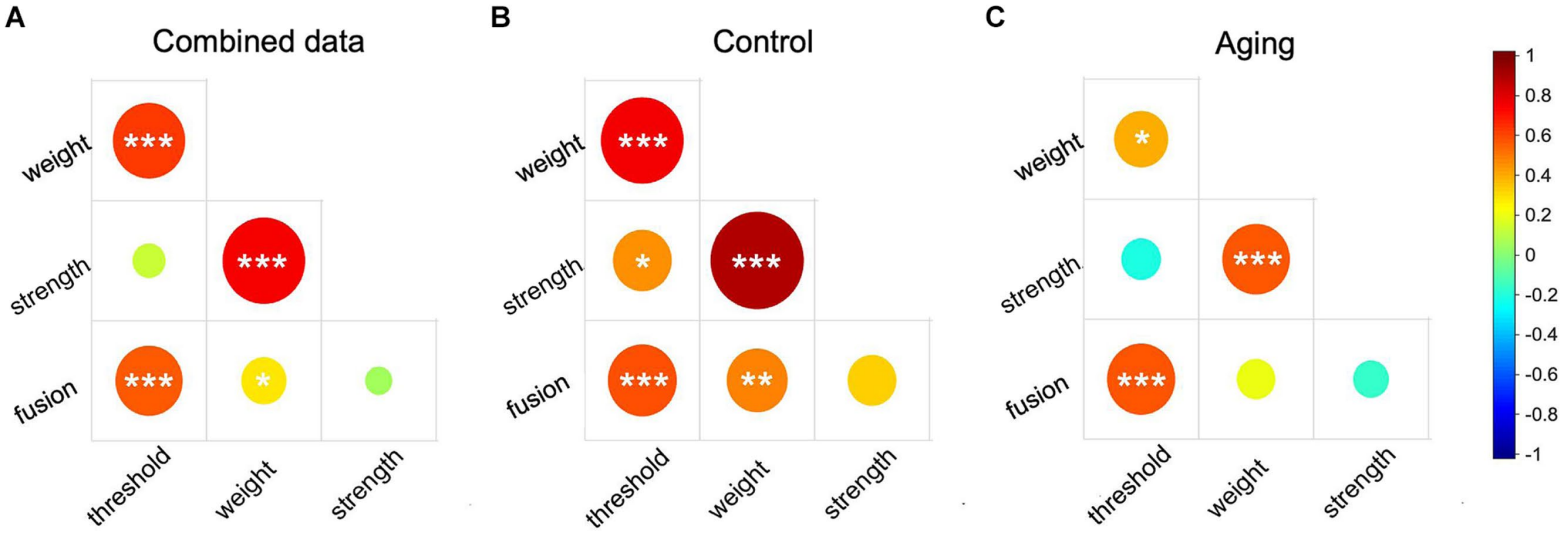
The correlation matrix of sensory eye imbalance in combined data **(A)**, the control group **(B)**, and the aging group **(C)**. Red color represents positive correlation and blue color represent negative correlation. The circle size represents the absolute correlation coefficient value. *p* ≤ 0.001 was marked with ***, 0.001 < *p* < 0.01 was marked with **, 0.01 < *p* < 0.05 was marked with *.

Based on our previous study (Wang et al., 2021), we regarded the interocular differences measured in a subset of our methods to each reflect a type of sensory eye imbalance. They are “threshold imbalance” (difference in contrast threshold between the two eyes), “fusion imbalance” (the balance point for when the two eyes contribute equally to the precept), “masking weight imbalance” (the difference in masking weight between the two eyes), and “masking strength imbalance” (the difference in masking strength between the two eyes). To further explore the relationships among these sensory eye imbalances, we performed correlation analysis in our “combined” data set (combining the data from the control and aging groups), the control group, and the aging group. The results of the combined data analysis and that from the control group were quite similar. The threshold imbalance was positively correlated with fusion imbalance and with the masking weight imbalance (*p* ≤ 0.001, for all, Figures 3A,B), and the weight imbalance was also significantly correlated with the fusion imbalance and with strength imbalance in both the combined data and the control group (*p* ≤ 0.032 for all, Figures 3A,B). In the aging group, we also found moderate correlations between the threshold imbalance and fusion imbalance (rho = 0.58, *p* < 0.001), and between the weight imbalance and strength imbalance (rho = 0.57, *p* = 0.001) (Figure 3C). The threshold imbalance was significantly correlated with the masking weight imbalance (rho = 0.40, *p* = 0.035), but the correlation coefficient reduced compared to control (rho = 0.77, *p* < 0.001) (Z = 2.08, *p* = 0.038) (Figures 3B,C). However, that the threshold imbalance and the strength imbalance were significantly correlated in controls (rho = 0.46, *p* = 0.014, Figure 3B) was not observed in the aging group (rho = −0.22, *p* = 0.270, Figure 3C). Significant correlations are provided in scatter plots as the Supplementary Figure S1.

Moreover, we are interested in whether these sensory eye imbalances could affect the stereopsis in different groups, and whether the stereoacuity assessed by varied stereo tests correlated with each other. We then analyzed the correlations between the absolute sensory eye imbalances and the stereoacuity in each data set. The use of the absolute imbalance is based on the idea that a strong preference for *either* eye may impair stereo vision (Webber et al., 2020). Neither the combined data nor the control group data showed significant correlations between any of the sensory eye imbalances and stereoacuity (*p* ≥ 0.094, Supplementary Figures S2A,B). However, in the aging group the stereoacuity measured by the Titmus was positively correlated with threshold imbalance (rho = 0.40, *p* = 0.034) and with weight imbalance (rho = 0.51, *p* = 0.006) (Supplementary Figure S2C and Supplementary Figures S1F,G). In addition, we found that the stereoacuity tested by Titmus was positively correlated with that tested by 4-C (rho = 0.35, *p* = 0.008) and TNO (rho = 0.37, *p* = 0.005) in the combined data (Supplementary Figure S2A and Supplementary Figures S1H,I), but these correlations were not observed in the control group (Supplementary Figure S2B). In the aging group, the stereoacuity assessed by Titmus and 4-C test were significantly correlated (rho = 0.38, *p* = 0.049, Supplementary Figure S2C and Supplementary Figure S1H). But these correlation coefficients were relatively moderate, which may be affected by the limitations of clinical stereo tests, the presence of outliers, and range effects.

## 4 Discussion

In the current study, we assessed the monocular contrast thresholds at a fixed moderate spatial frequency in both control and aging groups. Compared with the control, we found that the monocular contrast thresholds increased in the aging group (Figure 2A). The optical changes in the ocular refractive system that occur with age may cause a higher contrast threshold (Wolf and Gardiner, 1965; Owsley et al., 1983; Kersten et al., 1988). However, studies simulating these optical changes in young populations do not find similar increases in contrast threshold (Sloane et al., 1988; Whitaker and Elliott, 1992; Elliott et al., 2009). This has suggested that the neural processing changes in the aging brains also play an important role in their visual function changes. In our study, we recruited older subjects without manifest cataract or any ocular pathologic changes to reduce the effect of optical factors. Also, our stimuli relied on a moderate spatial frequency, which is below the range that visual function changes are more likely to attribute to optical factors (Hess and Woo, 1978; Elliott, 1987). Thus, we believe that the higher contrast threshold we observed in the aging group would be more associated with neural changes. Previous studies showed neural deterioration within the cerebral cortex (Raz et al., 2005; Yu et al., 2006; Smith et al., 2007), and neuron density decrease in both magnocellular and parvocellular pathways (Harman et al., 2000; Benedek et al., 2016), which may contribute to impaired contrast sensitivity in aging.

By using the modified two-stage model of Meese et al. (2006), we qualified and compared the relative contributions of suppression originating between each eye in the control and aging groups. Our results showed that the aging group exhibited a larger masking weight across the two eyes (Figure 2B), indicating that the eyes of the aging received a greater suppression effect from the contralateral eyes. This interocular suppression changes with age may be related to the impaired GABA inhibition in the older visual system. Previous studies demonstrated that the GABA level in the brain is a non-linear trajectory of the life span (Fagiolini and Hensch, 2000; Aufhaus et al., 2013). Several aged animal model studies reported that the proportion of GABAergic neurons is reduced in the V1 (Leventhal et al., 2003; Hua et al., 2008). However, this could not explain the findings from numerous psychophysical human studies. For example, aging populations have been showed to experience an increased central surrounding suppression (Karas and Mckendrick, 2011, 2012) and a slower binocular rivalry alteration rate (Ukai et al., 2003), which are associated with increasing GABA in their visual cortex (van Loon et al., 2013; Abuleil et al., 2019). More recently, one investigation with magnetic resonance spectroscopy measured the GABA concentration *in vivo*, and found that GABA level was higher in the primary visual cortex in older compared to younger adults (Pitchaimuthu et al., 2017). The greater interocular suppression weight we observed in the aging group may be attributed to this increased intracortical inhabitation.

In short, the aging group exhibited a lower gain but a higher weight of suppression relative to the control group. We subsequently compared the difference of net suppression (that is the overall effective interocular suppression strength) between the two groups. Based on our model, we can easily obtain the suppression strength by combining the input gain of one eye with the interocular suppression weight to the other eye. Interestingly, our results showed that the aging group had a comparable masking strength to the control group (Figure 2B). We propose that this may reflect a homeostatic mechanism that maintains the overall interocular suppression strength of the older group at the same level as the controls.

An analogy between the eye and person can be used to explain how it works, which is to imagine each eye as a person, with the suppression represented as each pushing the other with their hand. The suppression strength is the force that each person would feel from the push they receive. The gain would be the strength of the person pushing, with the weight of suppression being the amount of effort they are employing. So, an equal received “push” from the suppression could result either from a stronger person pushing with less effort (high gain, low weight of suppression) or a weaker person pushing with more effort (low gain, high weight of suppression). In our case, the aging group with lower input gain can be regarded as a weaker person and need to put more effect (higher weight of suppression) to induce a similar push force (suppression strength) as the controls (higher input gain and lower weight of suppression). Similar homeostatic compensation can be found in sensorimotor changes in aging, which demonstrated that a well-preserved vestibulo-ocular reflex function helps compensate the age-related degradation of postural control to prevent falls (Li et al., 2015; Luque et al., 2022).

Although we observed that the absolute balance point of the aging group was slightly greater than that of the controls, the difference between the two groups was not significant (Figure 2C). This is consistence with the study from Yan et al. (2021) who reported a similar balance point at a spatial frequency of 1 c/d between the older and younger adults. These results suggested that the two eyes had quite equal contributions at the binocular fusion process in both aging and young populations. Note that the balance points of aging individuals scattered more widely, giving a hint that the degradation of two eyes during the healthy aging process may be unequal.

In addition, we assessed the stereoacuity of aging and control groups by both classic clinical (Titmus and TNO) and recently-developed digital (4-C) stereo tests. Although different assessment methods can obtain different results (Vancleef et al., 2017; Tittes et al., 2019), our findings consistently showed a higher stereoacuity threshold in the aging group compared to the control group (Figure 2D). It has been reported that the sensory eye imbalance could significantly affect stereopsis (Webber et al., 2019; Wang et al., 2021). Unbalanced interocular inhibition has also been reported to reduce binocular depth perception (Xu et al., 2010, 2011). Han et al. (2019) demonstrated that the subjects with large magnitude of unequal interocular contrast inputs tended to have higher stereo threshold and longer stereo reaction time. We observed moderate correlations between the stereoacuity (tested by Titmus) and threshold imbalance, and between the stereoacuity (tested by Titmus) and masking weight imbalance in the aging group, which suggested that the alterations of the contrast input and weight of suppression may be the significant factors in impeding stereopsis in aging. It is reported that the clinical stereo tests contain the monocular and binocular stereo cues (Charman and Jennings, 1995; Fawcett, 2005; Chopin et al., 2019), which may bias the stereo threshold, although we carefully recheck the response of subjects with the tests rotated to improve the test efficiency. Further studies using psychophysical stereo tests with a larger sample are needed to confirm our findings.

Consistent with our previous results (Wang et al., 2021), we found that the threshold imbalance was positively correlated with the masking weight imbalance and with the fusion imbalance in combined data, aging group, and control group (Figure 3). But the correlation coefficient of aging group (rho = 0.4) was relatively small compared to that of the control group (rho = 0.77) (*Z* = 2.08, *p* = 0.038). This also supports our homeostatic hypothesis described above. In order to maintain the same net suppression effect as control, the interocular suppression weight of the older group increased to compensate for the lower effective input strength. As a consequence, the correlation coefficient of the older group was reduced compared to controls. We additionally explored the correlations between the masking weight and strength imbalances. Not surprisingly, since the masking strength imbalance was calculated partly from the same value used in the threshold imbalance and the masking weight imbalance, we found strong correlations between the masking weight imbalance and masking strength imbalance (Figure 3).

In this study, we conducted a comprehensive investigation of changes in binocular visual function with age. We explored correlations among different measures of sensory eye imbalance and stereopsis in the aging individuals. Our results showed that the monocular contrast threshold and stereopsis (assessed by both clinical and psychophysical tests) were impaired in the aging individuals. The normal correlations among the sensory eye imbalance (including threshold imbalance vs. strength imbalance, and weight imbalance vs. fusion imbalance) in young adults were disturbed in aging. These changes likely have a cortical origin. We also found evidence of a compensation mechanism that maintains a homeostatic balance in the interocular suppression as vision changes with age.

## Data availability statement

The raw data supporting the conclusions of this article will be made available by the authors, without undue reservation.

## Ethics statement

The studies involving humans were approved by Ethics Committee on Biomedical Research, West China Hospital of Sichuan University. The studies were conducted in accordance with the local legislation and institutional requirements. The participants provided their written informed consent to participate in this study.

## Author contributions

YS: Conceptualization, Data curation, Formal analysis, Investigation, Methodology, Validation, Writing – original draft, Writing – review & editing. XW: Conceptualization, Data curation, Formal analysis, Funding acquisition, Investigation, Methodology, Project administration, Software, Supervision, Validation, Writing – original draft, Writing – review & editing. ML: Data curation, Formal analysis, Resources, Writing – review & editing. AB: Methodology, Project administration, Software, Supervision, Writing – review & editing. LL: Conceptualization, Formal analysis, Investigation, Methodology, Project administration, Supervision, Validation, Writing – review & editing.
