# Supplementary figures and images for: Binocular function in the aging visual system: fusion, suppression, and stereoacuity
Source: Front Neurosci. 2024 Feb 28;18:1360619. doi: 10.3389/fnins.2024.1360619 (PMC10932981; doi:10.3389/fnins.2024.1360619)

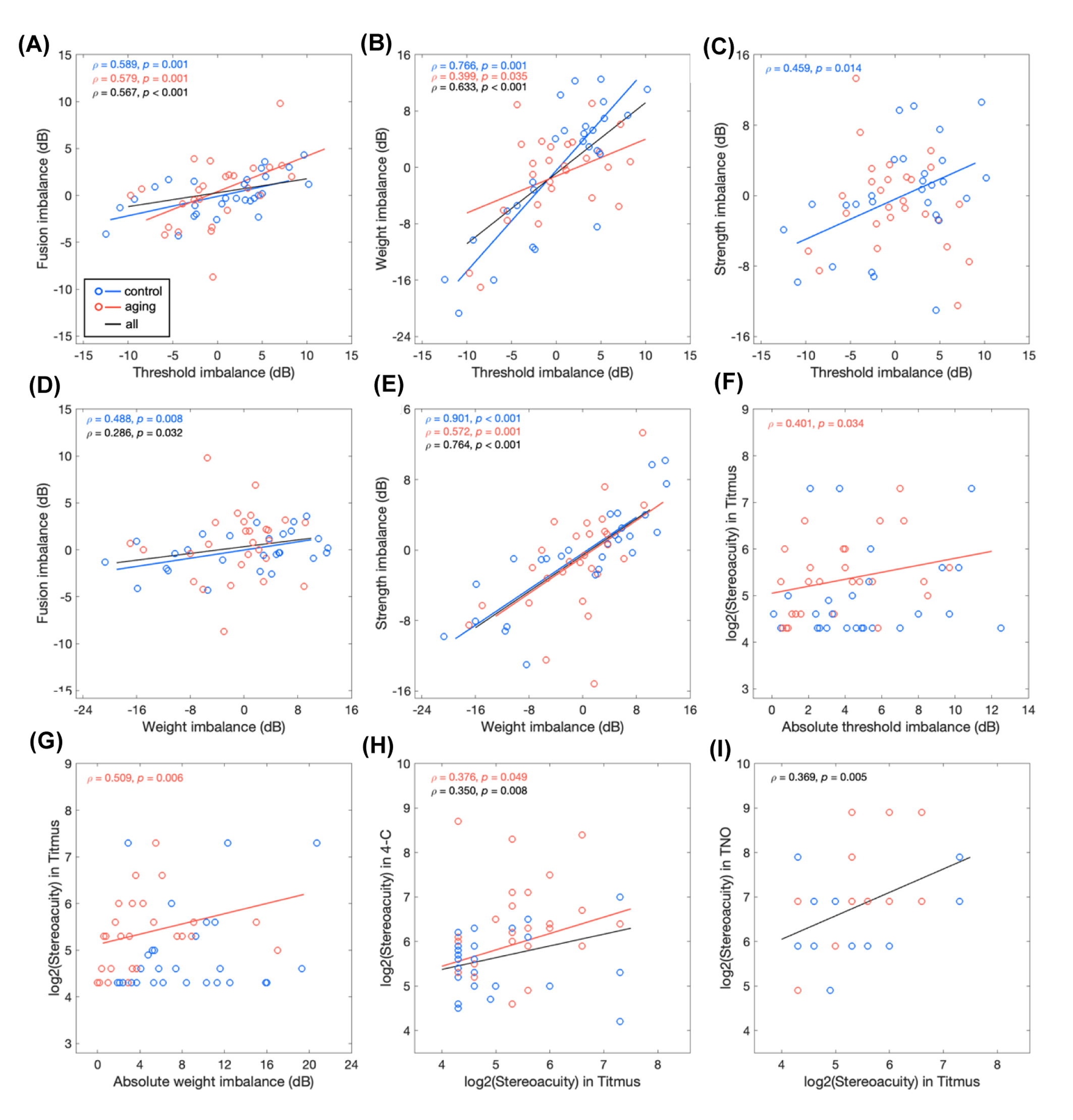

Supplement: Supplementary file 1 [file Image_1.TIF]

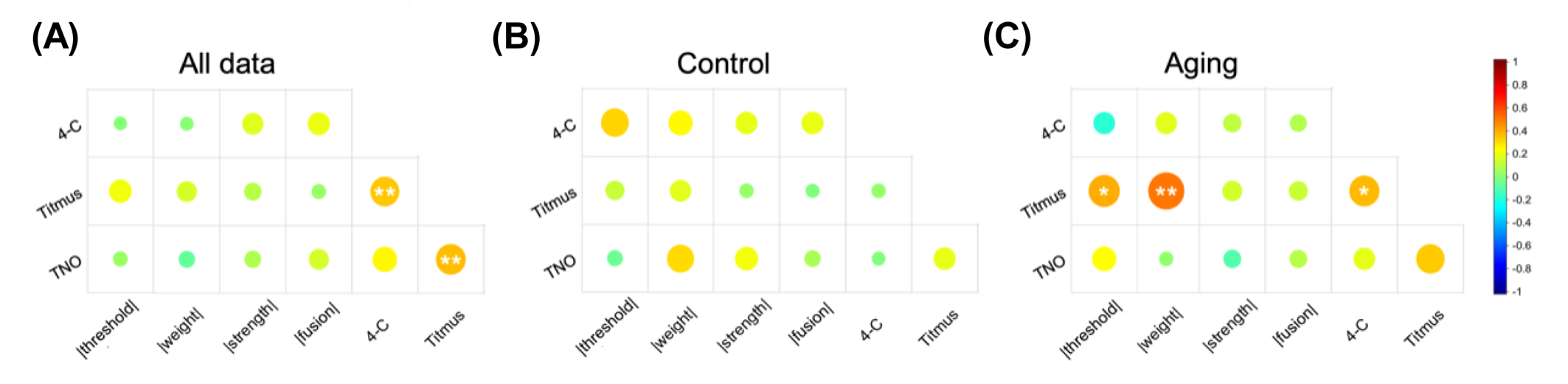

Supplement: Supplementary file 2 [file Image_2.TIF]
